# Supplementary material for: Structural insights into VRC01-class bnAb precursors with diverse light chains elicited in the IAVI G001 human vaccine trial
Source: Proc Natl Acad Sci U S A. 2025 Aug 11;122(33):e2510163122. doi: 10.1073/pnas.2510163122 (PMC12377726; doi:10.1073/pnas.2510163122)
Supplement: Supplementary file 1 — Appendix 01 (PDF) [file pnas.2510163122.sapp.pdf]

Supporting Information for

Structural insights into VRC01-class bnAb precursors with diverse light chains elicited in the IAVI G001 human vaccine trial

Xiaohe Lin[a], Christopher A. Cottrell[b,c], Oleksandr Kalyuzhniy[b,c,d], Ryan Tingle[b,c,d], Michael Kubitz[b,c,d], Danny Lu[b,c,d], Meng Yuan[a], William R. Schief[b,c,d,e,1], Ian A. Wilson[a,1]

[a] Department of Integrative Structural and Computational Biology, The Scripps Research Institute, La Jolla, California, USA.
[b] Center for HIV/AIDS Vaccine Development, The Scripps Research Institute, La Jolla, CA 92037, USA.
[c] Department of Immunology and Microbial Science, The Scripps Research Institute, La Jolla, CA 92037, USA.
[d] IAVI Neutralizing Antibody Center, The Scripps Research Institute, La Jolla, CA 92037, USA.
[e] Moderna Inc., Cambridge, MA, 02139, USA.
[1]To whom correspondence may be addressed. Email: wilson@scripps.edu; schief@scripps.edu.

ORCHID ID:
Xiaohe Lin: 0000-0001-6761-3897
Christopher A. Cottrell: 0000-0002-3364-3083
Oleksandr Kalyuzhniy: 0000-0002-5273-1951
Ryan Tingle: 0000-0003-2874-7010
Michael Kubitz: 0000-0002-3917-453X
Danny Lu: 0000-0002-2340-3377
Meng Yuan: 0000-0001-9754-4503
William R. Schief: 0000-0002-1120-0150
Ian A. Wilson: 0000-0002-6469-2419

Author Contributions: X.L., C.A.C., W.R.S. and I.A.W. designed research; X.L., R.T., M.K. and D.L. expressed and purified proteins; X.L. crystallized proteins; X.L. and M.Y. collected x-ray data, X.L. determined, refined and analyzed crystal structures; O.K. performed surface plasmon resonance; X.L., C.A.C., W.R.S. and I.A.W. analyzed data; X.L., C.A.C., W.R.S. and I.A.W. wrote the paper; and all authors reviewed the paper.

Competing Interest Statement: W.R.S. is an inventor on patents filed by Scripps and IAVI on the eOD-GT8 monomer and 60mer immunogens. W.R.S. is an employee and shareholder of Moderna, Inc. The other authors have no competing interest to declare.

Classification:
Major category: Biological Sciences: Biophysics and Computational Biology
Minor category: Biological Sciences: Microbiology

Keywords: Germline-targeting vaccine, VRC01-class antibodies, HIV CD4-binding site, X-ray crystallography, N276 glycan accommodation

This PDF file includes:

Figures S1 to S4
Tables S1 to S2
Data S1

Fig. S1. Conserved CD4bs engagement by antibody precursors and bnAbs. (A) Top and side views of the five VRC01-class bnAb precursor Fabs superposed onto the trimeric BG505 SOSIP structure (PDB: 6X9R) via alignment to eOD-GT8. This model illustrates the conserved CD4bs-directed binding angles. No contacts are observed between the precursor Fabs and the adjacent Env protomer. Antibody Fabs are colored as follows: G001-0087 (red), G001-58 (yellow), G001-59 (blue), G001-179 (pink), and G001-14 (green). (B) Top and side views of the VRC01-class bnAbs VRC01 (PDB: 3NGB, red), DRVIA57 (PDB: 5CD5, yellow), VRC23 (PDB: 4J6R, blue), and N6 (PDB: 5TE6, orange), superposed onto the trimeric BG505 SOSIP structure (PDB: 6X9R) via alignment to HIV gp120 core. Binding angles are conserved and similar to those in panel (A). No contacts are observed with adjacent protomers. (C) Epitope defined by contact residues (as in Fig. 1D) for bnAbs DRVIA57 (PDB: 5CD5), VRC23 (PDB: 4J6R) and N6 (PDB: 5TE6) complexed with HIV gp120 core. Residue positions contacted by all five precursors in (Fig 1D, left) are highlighted with a mesh. See also Fig. 1D for VRC01 epitope.

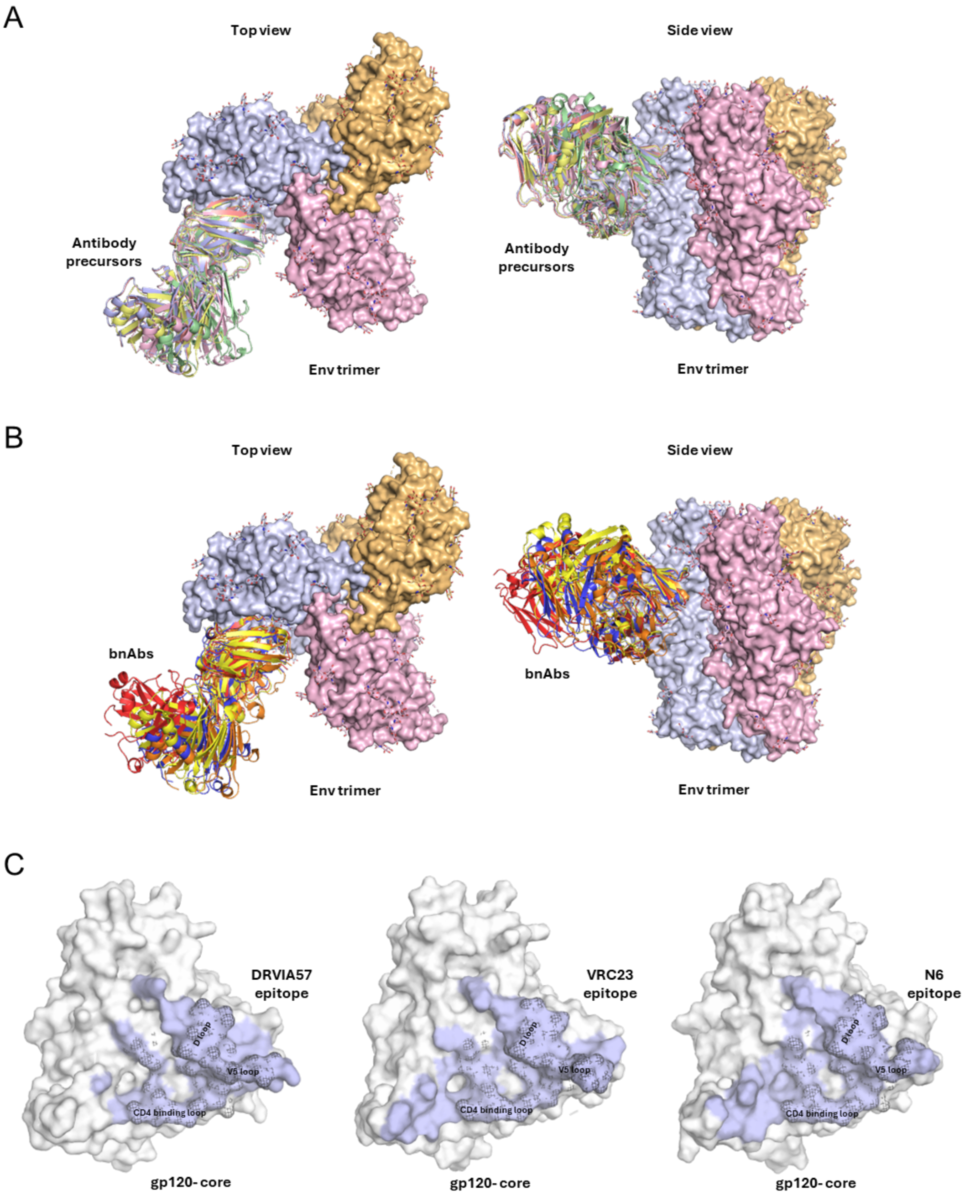

Fig. S2. Conserved heavy-chain interactions across antibody precursors. Zoomed-in view of the IGHV1-2 interaction (as in Fig. 2B) for four G001 antibody precursors (G001-14, G001-58, G001-59 and G001-179). Residues involved in hydrogen bonds are shown in sticks, and conserved hydrogen bonds in both antibody precursor and bnAb VRC01 are indicated by black dashed lines. All other hydrogen bonds are marked by yellow dashed lines.

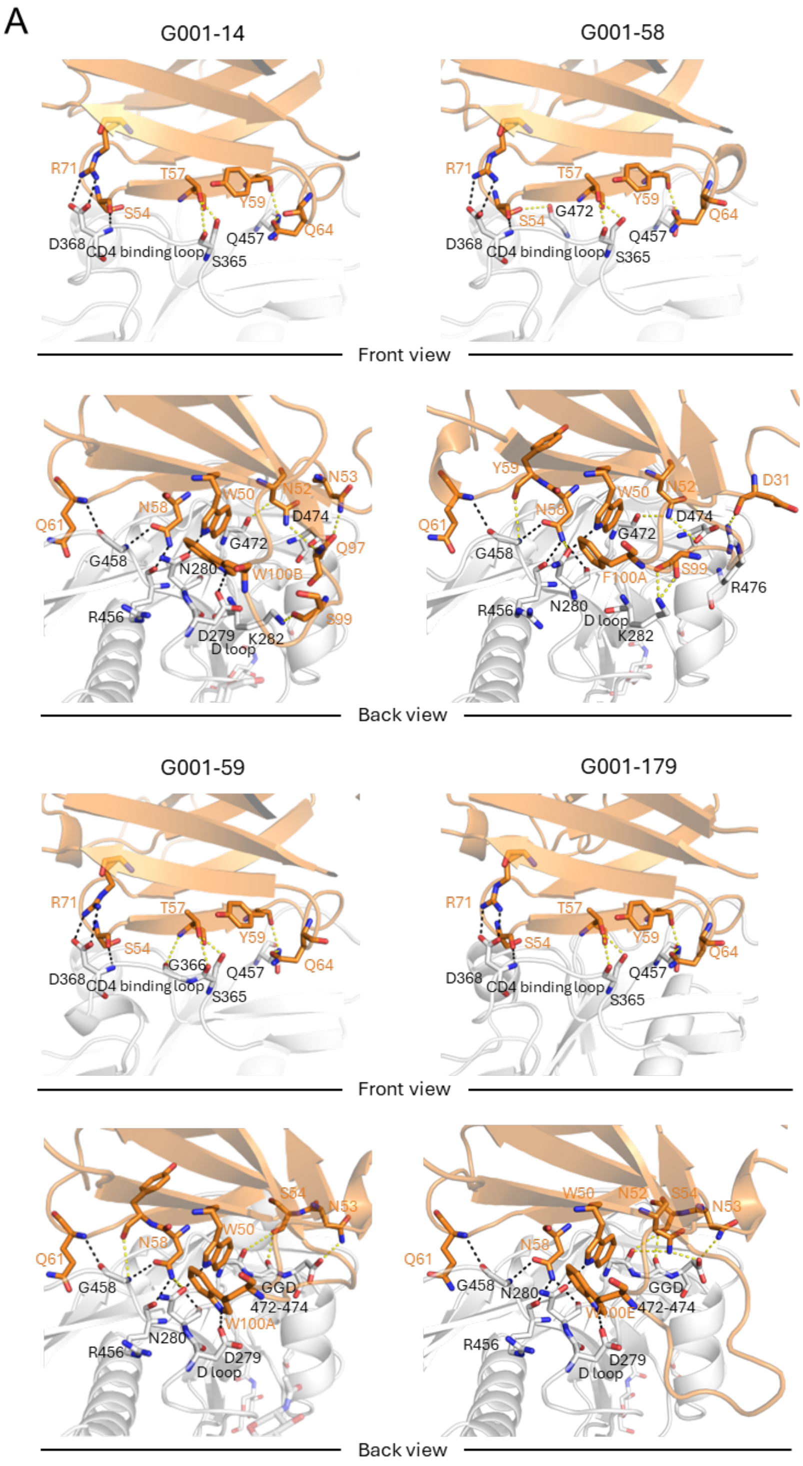

Fig. S3. Conserved positioning of the HCDR3 tryptophan/phenylalanine residue. Structures of HCDR3 loops from five G001-derived VRC01-class bnAb precursor antibodies (G001-0087, G001-14, G001-59, G001-58 and G001-179) and mature bnAb VRC01, centered on the conserved tryptophan or phenylalanine residue that interacts with residue D/N279 on Env. Although the HCDR3 loops vary in lengths, the conserved aromatic residue (Trp/Phe) is located at the same relative position with respect to D/N279 on gp120.

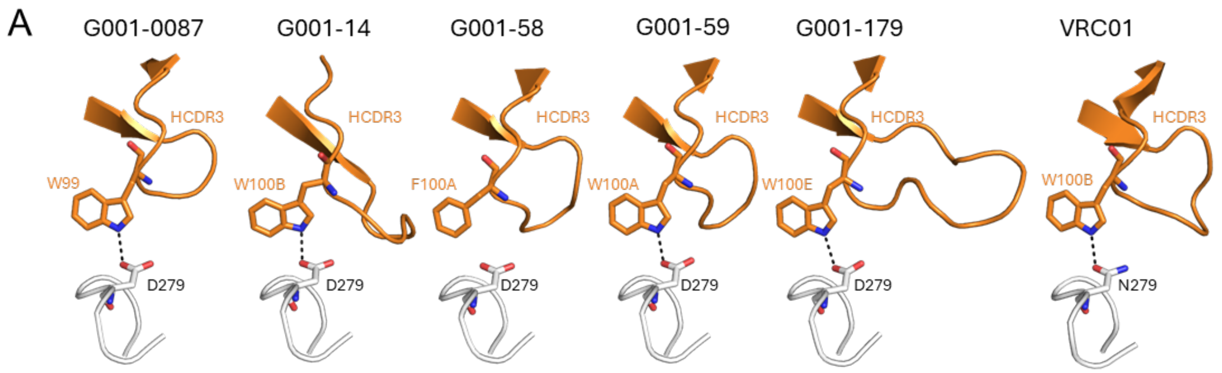

Fig. S4. Light-chain interactions across antibody precursors. Zoomed-in view of light-chain interactions (as in Fig. 3C) for four G001 antibody precursors. (G001-14, G001-58, G001-59 and G001-179). (Top: hydrogen bonds between the precursor light chain and antigen; bottom: steric constraints on LCDR3 imposed by antigen-proximal regions.) The five-amino-acid LCDR3 is shown in sticks to illustrate how it avoids steric clashes with adjacent regions including LCDR1, HCDR3, and the D and V5 loops of the antigen, which are shown in surface representation.

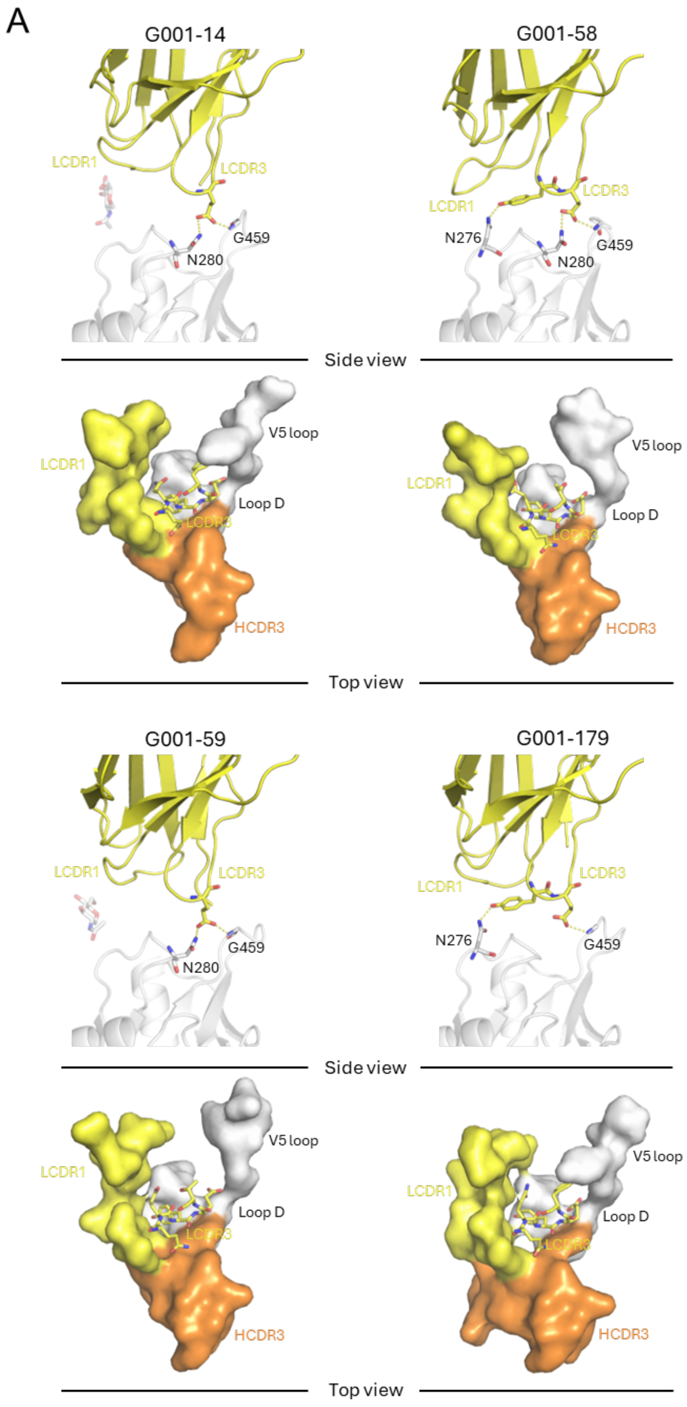

Table S1. Properties of the five VRC01-class bnAb precursors selected for structural analysis.

Antibody name	Timepoint	eOD-GT8 Kon	eOD-GT8 Koff	eOD-GT8 KD (M)	Specimen Type	Heavy V gene	Heavy D gene	Heavy J gene	HCDR3 length	Heavy V gene identity (aa)	Light V gene	Light J gene	HCDR3 length	Light V gene identity (aa)
G001_gp1_V10_VRC01c_2021-0087	week 16	4.20E+05	6.20E-05	1.50E-10	PBMC	IGHV1-2*04	IGHD3-10*01	IGHJ4*02	11	91.84%	IGKV3-20*01	IGKJ4*01	5	96.70%
G001_LD_07A_VRC01c_58	week 9	3.98E+05	1.74E-04	4.37E-10	PB	IGHV1-2*04	IGHD5-12*01	IGHJ4*02	13	93.88%	IGKV1-5*03	IGKJ1*01	5	98.90%
G001_LD_07A_VRC01c_59	week 9	3.94E+05	6.93E-04	1.76E-09	PB	IGHV1-2*04	IGHD6-13*01	IGHJ4*02	13	95.92%	IGKV3-15*01	IGKJ1*01	5	98.90%
G001_gp1_V10_VRC01c_179	week 16	4.50E+05	5.90E-05	1.30E-10	PBMC	IGHV1-2*02	IGHD2-15*01	IGHJ4*02	17	93.81%	IGKV1-33*01	IGKJ3*01	5	98.90%
G001_LD_V05_VRC01c_14	week 3	2.10E+05	7.60E-05	3.70E-10	FNA	IGHV1-2*04	IGHD6-19*01	IGHJ4*02	14	96.94%	IGKV1-33*01	IGKJ4*01	5	98.90%

Table S2. Data collection and refinement statistics.

**Data collection**	eOD-GT8-mingly + G001-0087-Fab	eOD-GT8-mingly + G001-58-Fab	eOD-GT8-mingly-N276 + G001-59-Fab	eOD-GT8-mingly + G001-179-Fab	eOD-GT8-mingly-N276 + G001-14-Fab
Beamline	APS 23-ID-B	SSRL 12-1	SSRL 12-1	SSRL 12-1	SSRL 12-1
Wavelength (Å)	1.0332	0.9795	0.9795	0.9795	0.9795
Space group	I 2 2 2	C 2 2 2_1	I 2 2 2	P 1	C 1 2 1
Unit cell parameters					
a, b, c (Å)	102.5, 136.171, 152.944	110.1, 174.9, 93.6	102.7, 134.0, 154.0	49.2, 65.9, 122.1	261.1, 75.5, 158.1
α, β, γ (°)	90, 90, 90	90.0, 90.0, 90.0	90.0, 90.0, 90.0	89.9, 97.0, 90.0	90.0, 92.4, 90.0
Resolution (Å) [a]	50.00 - 2.60 (2.64 - 2.58)	50.00-2.80 (2.87-2.80)	50.00-2.54 (2.58-2.54)	50.00-1.97 (2.00-1.94)	50.00-2.81 (2.86-2.79)
Unique reflections [a]	33,387 (1,503)	22,255 (1,109)	34,890 (1,696)	96,860 (4,358)	75,138 (3,714)
Redundancy [a]	10.5 (4.0)	12.8 (12.2)	11.4 (6.1)	3.3 (2.8)	6.8 (6.9)
Completeness (%) [a]	99.3 (90.9)	99.1 (99.4)	99.0 (98.3)	89.6 (80.8)	98.4 (99.2)
$<I/\sigma_I>$ [a]	12.3 (1.1)	8.3 (1.0)	14.5 (1.0)	5.8 (2.1)	7.7 (1.3)
R_{sym} [b] (%) [a]	25.9 (115.9)	53.7 (686.4)	16.5 (191.4)	26.6 (139.3)	31.4 (346.6)
R_{pim} [b] (%) [a]	7.6 (49.9)	14.9 (194.8)	4.8 (72.5)	14.3 (78.5)	11.9 (131.3)
$CC_{1/2}$ [c] (%) [a]	98.0 (42.9)	98.7 (41.1)	99.7 (39.3)	95.0 (32.3)	97.8 (31.1)
**Refinement statistics**					
Resolution (Å)	47.74 - 2.58	47.44-2.80	38.50-2.54	26.28-1.94	38.18-2.79
Reflections (work)	33,110	21,156	34,672	96,730	74,974
Reflections (test)	1,631	1,070	1,668	5,082	3,808
R_{cryst} [d] / R_{free} [e] (%)	23.8 / 25.3	21.3/24.9	20.8/23.0	21.3/24.8	21.4/25.4
No. of atoms	4,696	4,541	4,719	10,722	18,474
Antigen	1,288	1,288	1,284	2,576	5,136
Fab	3,228	3,225	3,259	6,448	13,036
Glycan	28	28	42	56	168
Solvent	152	-	134	1,642	134
Average *B*-values ($Å^2$)	48	63	58	23	67
Antigen	56	69	66	22	61
Fab	45	60	54	23	69
Glycan	73	96	94	31	88
Solvent	43	-	50	25	51
Wilson *B*-value ($Å^2$)	44	64	56	18	61
**RMSD from ideal geometry**					
Bond length (Å)	0.003	0.003	0.002	0.007	0.004
Bond angle (°)	0.55	0.50	0.54	0.92	0.71
**Ramachandran statistics (%)**					
Favored	96.4	95.7	97.8	96.6	96.6
Outliers	0.00	0.34	0.17	0.17	0.13
**PDB ID**	9OAO	9OAP	9OAQ	9OAR	9OAS

Data S1. SPR and sequence data of VRC01-class bnAb precursors isolated from the IAVI G001 vaccine trial.
